# ICDXML: enhancing ICD coding with probabilistic label trees and dynamic semantic representations

**DOI:** 10.1038/s41598-024-69214-9

**Published:** 2024-08-07

**Authors:** Zeqiang Wang, Yuqi Wang, Haiyang Zhang, Wei Wang, Jun Qi, Jianjun Chen, Nishanth Sastry, Jon Johnson, Suparna De

**Affiliations:** 1 Department of Computing, Xi’an Jiaotong Liverpool University, Suzhou, 21500 China; 2 School of Computer Science and Electronic Engineering, University of Surrey, Surrey, GU2 7XH UK; 3 Department of Computer Science, University of Liverpool, Liverpool, L69 3BX UK; 4 UCL Social Research Institute, University College London, London, WC1E 6BT UK

**Keywords:** Natural language processing, ICD coding, Extreme multi-label classification, Few-shot learning, Medical knowledge representation, Computer science, Computational science

## Abstract

Accurately assigning standardized diagnosis and procedure codes from clinical text is crucial for healthcare applications. However, this remains challenging due to the complexity of medical language. This paper proposes a novel model that incorporates extreme multi-label classification tasks to enhance International Classification of Diseases (ICD) coding. The model utilizes deformable convolutional neural networks to fuse representations from hidden layer outputs of pre-trained language models and external medical knowledge embeddings fused using a multimodal approach to provide rich semantic encodings for each code. A probabilistic label tree is constructed based on the hierarchical structure existing in ICD labels to incorporate ontological relationships between ICD codes and enable structured output prediction. Experiments on medical code prediction on the MIMIC-III database demonstrate competitive performance, highlighting the benefits of this technique for robust clinical code assignment.

## Introduction

In the modern era of big data and advancing health technologies, accurate classification and documentation of medical diagnoses and procedures are critical for effective healthcare management and data-driven decision-making. The International Classification of Diseases (ICD) coding system serves as a standardized framework for precisely categorising the wide range of medical conditions and interventions encountered in healthcare settings^1^. With the widespread adoption of electronic health records (EHRs), detailed patient information is now captured in unstructured clinical notes. These notes, such as discharge summaries, contain valuable data on patients but are often lengthy and written in medical domain language. Manual clinical coding in EHR is thus time-consuming, labor-intensive, and prone to inconsistent coding between healthcare providers^2^. In such cases, there is a growing need for automated approaches that can reliably extract information from clinical notes and accurately assign standardized ICD codes.

Natural language processing (NLP) methods hold promise for developing high-throughput systems to automate ICD coding from unstructured EHR text^3^. Recent studies have shown that pre-trained language models (PLMs), such as BERT^4^, Alpaca^5^, and Llama^6^, have shown great success in capturing domain-specific knowledge of medical language, leveraging the contextual information of sentences and adapting to different medical tasks^7,8^. These models have been widely employed in the research community for supervised learning tasks involving EHR data. For example, BERT-based PLMs like ClinicalBERT^9^ and BlueBERT^10^ have been fine-tuned and evaluated on medical diagnosis prediction based on patient notes, which can analyze unstructured clinical text to identify and classify reported symptoms, tests, and assessments into diagnostic codes. More recently, PLMs have also been adopted for automating ICD code assignment from EHRs^11^. Such models can be trained on notes labelled with relevant codes to learn associations between clinical language and standardized billing codes. Fine-tuned transformer models have achieved state-of-the-art results for multi-label ICD coding across various datasets, and their contextual representations are able to capture ambiguities in medical language compared with traditional NLP approaches.

While PLMs have shown their capabilities for learning generalisable representations of medical language, automated ICD coding still remains highly challenging due to several factors^12,13^. Firstly, the label space consisting of thousands of ICD codes presents modelling difficulties. Predicting from such a large set of codes in a multi-label setting is considerably more complex compared to single-label categorization; secondly, the label distribution in EHR is extremely imbalanced, also known as the long-tail problem, where the majority of ICD codes have only a few positive examples. This issue makes it difficult to learn robust patterns for rare codes. Finally, the fragmented nature of EHR notes makes extracting signals for each code label difficult. Relevant text for a particular code may be scattered throughout a lengthy note. Identifying these scattered semantic signals in a unified manner is non-trivial.

Given the complex challenges posed by multi-label classification at scale, it is imperative to incorporate external knowledge, such as taxonomic hierarchy and textual definitions for the automated ICD coding task. Specifically, the taxonomic hierarchy between ICD codes provides useful semantic signals, such as parent-child constraints and code similarity clusters that can effectively reduce spurious predictions. Textual definitions of each ICD code offer important contextual details, and incorporating these as supplementary descriptors can help models better characterize the intended meaning of codes. In this work, we present an effective ICD coding model called ICDXML, which utilizes a label tree to integrate both structural and textual knowledge for the task of extreme multi-label classification. By jointly leveraging these complementary knowledge types, our framework aims to improve context-aware prediction of relevant codes from sparse and imbalanced EHR data. Experiments on MIMIC-III datasets^14,15^ demonstrate the ability of our integrated approach to advance the state-of-the-art in automated ICD coding through the fusion of external domain knowledge into deep neural architectures.

## Related work

Automated ICD coding using NLP techniques to assign standardized codes to discharge summaries in EHR has been extensively studied and gained significant research attention. Earlier approaches relied heavily on manually curated rules such as predefined inclusion and exclusion criteria^16,17^. However, these rule-based systems required extensive expert effort to engineer effective rules. They also lacked the flexibility to handle medical language and required constant updating.

Machine learning methods, such as logistic regression^18^ and support vector machines^19^, have been utilized to learn statistical associations between text and codes. However, these methods struggled to scale to the high dimensionality of thousands of ICD codes and the multi-label nature of assigning multiple codes per note.

Recent breakthroughs in deep learning have shown significant potential for automated ICD coding. Previous studies^20,21^ have utilized recurrent neural networks like Long Short-Term Memory Networks (LSTMs), Convolutional Neural Networks (CNNs) and Graph Neural Networks (GNNs). These architectures have demonstrated an ability to identify higher-level abstract features within the medical text and capture semantic context effectively, particularly across lengthy clinical notes. Particularly, for GNNs, the structural information and dependencies in the medical graph can be better captured, allowing the model to learn the relationships among the symptoms and diseases, etc.^22,23^ Based on these architectures, researchers have also incorporated attention-based mechanisms^15,24^ into model architectures, enabling selectively focusing on pertinent segments of the input text to enhance the precision of code predictions. Furthermore, the integration of label graphs allows for explicit modelling of coding hierarchy and co-occurrence relationships between codes, contributing to more accurate code assignments^25^. Additionally, multitask learning has been explored as a beneficial approach for ICD coding^26,27^. By jointly training on multiple related tasks, such as ICD and Clinical Classification Software (CCS) code prediction, multitask learning schemes can effectively transfer ICD code association knowledge from the auxiliary task branch, leading to improved performance in ICD code prediction.

Another significant development is the adoption of transfer learning techniques, where large-scale medical corpora are pre-trained using transformer-based language models before fine-tuning them for specific ICD coding tasks^9,28,29^. This approach has gained popularity due to its capacity to leverage the knowledge contained within extensive medical datasets, ultimately leading to improved performance in automated ICD coding systems. Moreover, several studies have explored using pre-trained generative models instead of discriminative models by reframing text classification as a text generation problem^30^. The generated text can then be mapped to the corresponding ICD code.

## ICDXML

### Input representation

**Figure 1:**
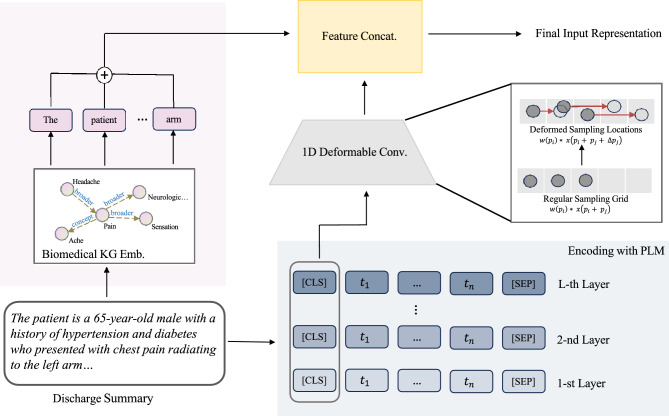
Input representation generation for the discharge summary. In the 1D deformable convolution block, we illustrate the sampling process using regular (lower) and deformable (upper) convolution, respectively. The dark grey points represent the sampling locations, while the red arrows indicate the offsets in the deformable convolution.

To represent the discharge summary, we begin by encoding the textual data with the PLM and integrating this representation with our proposed one-dimensional deformable convolutional structure. We concatenate the features from the PLMs and knowledge graph embeddings to enhance the quality of our representations. An overview of the input representation generation for discharge summaries is illustrated in Fig. 1.

#### Pre-trained language model encoding

One typical paradigm for text representation is to use PLMs to extract vector representations of text. Our model follows this design principle and further considers that the length of EHR texts often exceeds the maximum input length limit of models like BERT. Therefore, we use ClinicalLongformer^31^, a PLM for long texts pre-trained on medical corpora, to encode the discharge summaries and generate corresponding semantic representations. In previous research, the vector of the CLS position from the last layer of the PLM is commonly used as the representation of the entire input. However, recent research has shown that using the average of the last layer and first layer hidden states can achieve better results. We conjecture that because the bottom hidden state retains more abundant textual feature information, while the top hidden state contains more advanced semantic abstractions, combining both can provide a more comprehensive text representation, thereby enhancing the performance of downstream tasks. Specifically, given an input token sequence $t=\{\text {[CLS]}, t_1, t_2,..., t_n, \text {[SEP]}\}$, we obtain a series of contextualized hidden state representations at *l*-th layer, $\textbf{h}^l=[h_1^l, h_2^l,...,h_m^l]$, instead of the CLS token, where *m* denotes the dimensionality of the hidden states. We use the representations of the last hidden state $h_i^L$ and the first hidden state $h_i^1$ to calculate the average as the *i*-th element in the final encoding vector for the text:

$$\begin{aligned} h_i = \frac{1}{2}(h_i^L + h_i^1), \quad i = 1,2,\ldots ,m \end{aligned}$$

In this way, the representation integrates bottom-layer semantic features and high-level semantic abstractions, which will provide a high-quality text representation for downstream tasks.

#### Deformable convolutions for hidden states fusion

To enhance the representation capability of our model, we introduce the integration of deformable convolutional structures, which dynamically fuse hidden states from the PLM. This concept, rooted in the computer vision domain, initially featured two-dimensional deformable convolutional networks.

CNNs have achieved tremendous success in image processing by extracting local features through sliding convolution kernels over the input image. The convolution stride, which determines the stride at which the kernel slides, is an important parameter. A fixed stride ensures consistency between features extracted at different positions but makes it difficult to extract high-level semantic features. Deformable convolutions, proposed by Dai et al.^32^, were initially designed for computer vision to handle irregular objects by adjusting kernel shape to fit receptive fields.

In natural language processing, CNNs have also been widely applied to tasks like text classification and named entity recognition, usually with a fixed stride, which struggles to capture long-distance dependencies. To address this limitation, we introduce one-dimensional deformable convolutions to perform dynamic fusion of the hidden states to achieve more dynamic combinations. Like the two-dimensional counterparts, one-dimensional deformable convolutions adaptively adjust kernel sampling, resulting in richer combinations and semantic representations. Dynamic convolutions are expected to enhance the discriminative capacity of representations, which is likely to have a positive impact on downstream performance. A typical operation in a convolutional neural network can be expressed as:

$$\begin{aligned} o(p_i) = \sum _{p_j \in \mathcal {R}} w(p_i) * x(p_i + p_j) \end{aligned}$$

where $o(p_i)$ represents the value of the output feature map at position $p_i$, $w(p_i)$ denotes the weight of the convolution kernel at relative position $p_i$, $x(p_i + p_j)$ denotes the value of the input feature map at position $p_i + p_j$ and $\mathcal {R}$ denotes the receptive field of the convolution kernel, i.e. in the case of 1D convolutional neural network with a kernel size of 3, $\mathcal {R} = \{-1, 0, 1\}$. For deformable convolution, an offset $\Delta p_j$ is added for each sampling location based on the standard convolution operation:

$$\begin{aligned} o^\prime (p_i) = \sum _{p_j \in \mathcal {R}} w(p_i) * x(p_i + p_j + \Delta p_j) \end{aligned}$$

where $\Delta p_j$ is jointly learned from the input feature map and another convolutional layer, typically resulting in decimal values. Hence, linear interpolation is required to compute $x(p_i + p_j + \Delta p_j)$. In this way, the sampling locations of the convolutional kernel are no longer restricted to fixed grids but can be deformed according to the content of the input feature to obtain a more flexible representation. We use this proposed one-dimensional deformable convolution to dynamically learn the fusion weights of different layer states in the first dimension, i.e.

$$\begin{aligned} \textbf{h}^{\prime } = \textrm{DCN1d}(\textbf{H}; \textbf{w}) \end{aligned}$$

where $\mathrm {DCN1d(\cdot )}$ is the one-dimensional deformable convolution operation, $\textbf{H}$ is the set of all hidden states from PLM at the CLS position, i.e. $\textbf{H}=\{\textbf{h}^1, \textbf{h}^2,..., \textbf{h}^L\}$, $\textbf{h}^{\prime }$ stands for the new state after fusion with the deformable convolution, and $\textbf{w}$ is the learnable offset that controls the dynamic range of fusion.

#### Biomedical knowledge integration

Existing research has shown that injecting external knowledge into language models can greatly improve performance on lengthy text classification by introducing external cues while enhancing model sensitivity to keywords. A key challenge in this task is maintaining responsiveness to keywords without overly mixing them into abstract semantic information. To address this challenge, in addition to representations from the PLM, we also encode the discharge notes using pre-trained biomedical knowledge graph embeddings^33^ from Medical Subject Headings (MeSH)^34^ for knowledge injection. When merging the PLM and external knowledge representations in the biomedical domain, we note that the two representations, generated from meaningful semantic space and knowledge graph space, respectively, can be seen as heterogeneous concatenation.

Recent studies, such as BERT-flow^35^ and BERT-whitening^36^, have revealed the anisotropy of sentence embeddings generated by PLMs. Specifically, in the PLM vector space, low-frequency words are distributed sparsely, while high-frequency words are densely clustered. This is in contrast to knowledge graph embeddings, which are typically isotropic with uniform coverage of the vector space. The anisotropy arises from the objective of PLM to focus on learning predictive relationships between frequently co-occurring words. Therefore, simply concatenating the sentence representations from PLM with biomedical knowledge graph embeddings results in combining non-overlapping spaces with very different geometric shapes. To effectively integrate these heterogeneous representations, we propose borrowing techniques from multimodal factorized fusion^2^ to project vectors to a shared space before combining information, i.e.

$$\begin{aligned} \textrm{MFB}(\textbf{h}^\prime , \textbf{v}) = \textbf{U} * (\textbf{P}_{h^\prime } * \textbf{h}^\prime ) \otimes (\textbf{P}_v * \textbf{v}) \end{aligned}$$

where $\mathrm {MFB(\cdot )}$ is the Multi-modal Factorized Bilinear (MFB) operation, $\textbf{v}$ is the feature from the biomedical knowledge graph space, $\textbf{P}_{h^\prime }$ and $\textbf{P}_v$ are projection matrices for $\textbf{h}^\prime$ and $\textbf{v}$, respectively, $\otimes$ represents the outer product and $\textbf{U}$ is the learnable weight matrix.

### Output prediction

Our approach performs multi-label classification by encoding the input discharge summary *s* into a representative vector $\textbf{s}$ and leveraging a tree-based classifier for large output spaces. For small label sets, independent classifiers are trained on a per-label basis. However, when the number of labels $|\mathcal {Y}|$ exceeds the extreme classification threshold, we construct a hierarchical label tree *T* over the output space $\mathcal {Y}$ to exploit correlations and dependencies between ICD codes. The classification threshold was set as a group of candidate values by taking into account the number of potential labels for each text. The classification threshold that achieved the best performance on the validation set among the candidate values was chosen as the threshold for the test set. A specialized model $f_\theta$ with tree-structured classifiers is trained to capture these relationships. During prediction, $f_\theta$ takes the input representation $\textbf{s}$ and label hierarchy *T*, traversing the tree in a top-down manner to output predicted codes $\hat{y}$. This allows the model to scale effectively to large label spaces while retaining the output space structure. The overall process can be formalized in Algorithm 1

**Algorithm 1:**
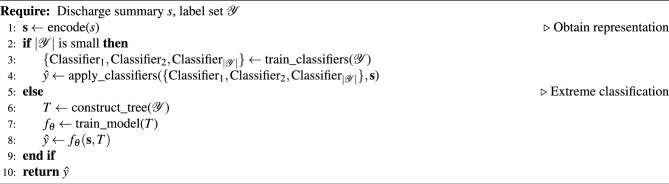
Multi-Label Text Classification

We will introduce the bottom-up label tree construction and top-down hierarchical label prediction in the following subsections.

#### Bottom-up probabilistic label tree construction

Currently, the best solutions for multi-label text classification are mostly label-wise and trainable on small label sets. However, dedicated classification heads for each label are infeasible with extremely large label sets. Therefore, inspired by the study of You et al.^37^, we adopt an extreme multi-label classification approach, computing text representations against a probabilistic label tree to predict probabilities for each label.

**Figure 2:**
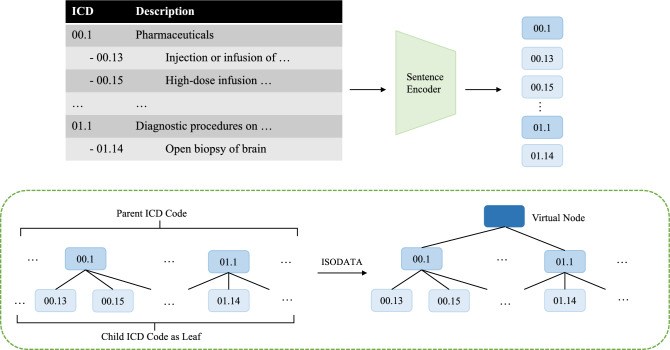
Overall workflow for the probabilistic label tree construction.

Unlike existing extreme classification datasets, the potential tree structure in ICD codes is definite, with certain diseases belonging to the same entry. However, diseases in ICD lack direct relationships, and the hierarchy can be seen as a forest structure. Hence, we propose a bottom-up approach (illustrated in Fig. 2) to construct a probabilistic label tree, incorporating the ICD forest into the tree.

In the initial step, we first retrieve all text descriptions for each ICD code from the MIMIC-III database. We then obtain vector representations of these descriptions using sentence transformers. We treat ICD codes as the terminal or leaf nodes of our taxonomy. Parent nodes are introduced to encapsulate ICD codes that share common parent descriptions. Notably, individual diseases devoid of parent nodes are considered to be self-contained parents in this hierarchical structure. In the following step, we initiate the hierarchical clustering process for all parent nodes. The clustering technique employed here is the Iterative Self-Organizing Data Analysis Technique (ISODATA)^38^, utilizing cosine similarity as the distance metric. Hyperparameters are thoughtfully controlled to strike a balance between the number of nodes beneath a parent and the overall breadth and depth of the resulting tree structure. We then consider the iterative refinement of the hierarchical structure. Specifically, this procedure is performed iteratively until the predetermined target tree depth is reached. The predetermined depth of the tree followed the common design logic for classification trees of being shallow and wide^37,39^. In this case, the depth of the classification tree was set to 4. At each iteration, parent-child relationships and the overall hierarchical arrangement are refined to ensure the attainment of the desired tree depth.

#### Top-down hierarchical label prediction

In the prediction stage, improving computational efficiency is critical since label tree-based inference entails large-scale matrix operations. However, the hierarchical structure of the labels can be leveraged to enhance predictive efficiency. Our model implements a dedicated, fully connected classifier head for each layer of the label tree. This further fuses the knowledge inputs and mixed text representations from the PLM into a consolidated vector space adapted to each tree layer. Fixing the label tree weights during prediction improves adaptability to the distinct levels of the hierarchy.

We first utilize a classifier head to generate text representations for the discharge summary and obtain probabilities for each possible node at the top. To limit the search space, when moving to the next layer of nodes, we only consider the top-*C* highest probability nodes from the current layer as candidates for splitting subtrees from the overall hierarchy. This recursive selection of the most likely child nodes prunes unlikely branches. We recursively execute this node prediction and candidate selection, traversing down the tree until it reaches the deepest layer of leaf nodes. Final code predictions are generated by thresholding the predicted probabilities at the leaves.

This greedy top-down traversal enables structured prediction over the output space by recursively focusing on the most probable child subtrees. Pruning subtrees with low predicted probability at higher layers narrow the search area, allowing scalability over large output hierarchies. The pseudo-code listed in Algorithm 2 summarizes the prediction with the constructed label tree.

**Algorithm 2:**
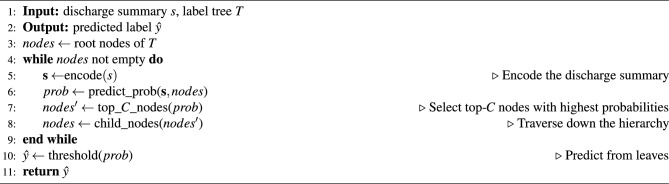
Prediction with Label Tree

## Results and evaluation

### Experimental setup

#### Dataset

Our study utilizes the MIMIC-III clinical text corpus containing de-identified discharge summaries from real hospitalizations, annotated with ICD-9 codes by medical experts. We preprocess the text in the following steps: First, de-identification tokens inserted to protect patient privacy were removed. Next, all characters apart from punctuation and alphanumerical content were replaced with white space to standardize the format and improve model processability. Finally, extraneous white space was stripped to streamline the text sequences, enhancing computational and memory efficiency for downstream models.

Given computational requirements, earlier approaches truncated summaries to 4,000 words. We employ the ClinicalLongformer^31^, which uses token-level inputs, enabling longer sequence modelling. Summaries are truncated at 8,192 tokens - empirically observed to approximate a 2 : 1 word-to-token ratio. Since ICD-9 codes relate to subjective note sections, we retain relevant segments for overlong summaries, discarding nonsalient discharge details. This comprehensive dataset is termed MIMIC-III-FULL. For predicting frequent codes, the data is filtered to samples with at least one of the 50 most common labels, using standard splits for comparison. This subset is denoted MIMIC-III-50. Descriptive statistics are tabulated in Table 1.

**Table 1:** Statistics of datasets

Dataset	# ICD codes	# Avg words	# Training set	# Testing set	Label type
**MIMIC-III-FULL**	8,922	1,485	47,724	3,372	Full code set
**MIMIC-III-50**	50	1,530	8,067	1,730	Top 50 frequent codes

#### Implementation details

To inject external knowledge into the model, we utilize word-level embeddings from BioWordVec^33^ for keyword representation. These biomedical knowledge graph embeddings are concatenated to the representations from the pre-trained language model to provide additional external cues. The BioWordVec embeddings contain complementary knowledge about term associations beyond what is captured solely from the discharge summary text. Moreover, to construct the label tree for prediction, we encode the textual ICD code descriptions using BioSimCSE-BioLinkBERT-BASE^40^. During the top-down search on the label tree, the number of highest probability nodes (*C*) from each layer is set to 20. To demonstrate the encoding of ICD codes and descriptions, sample text can be constructed as follows: “*The ICD number is [ICD Id]. The content is [ICD Text Description]*”, where *[ICD Id]* represents the numeric disease code, and *[ICD Text Description]* denotes the textual definition and description for that particular code. This encoding scheme concatenates the unique identifier with the associated descriptive content for each ICD entity.

We trained our models using PyTorch 1.9.0 and the Transformers library 4.11.3. All experiments were conducted on a cluster of 3 NVIDIA V100 GPUs, each with 32GB of memory. We employed Distributed Data Parallel (DDP) training to optimize resource utilization and accelerate the training process. We demonstrate the hyperparameters and training settings for fine-tuning in Table 2.

**Table 2:** Hyperparameters and training settings

Parameter	Value
Optimizer	AdamW
Initial learning rate	1e-4
Learning rate schedule	Linear warmup (10% steps) followed by linear decay
$\beta _1$, $\beta _2$	0.9, 0.999
$\epsilon$	1e-8
Weight decay	0.01
Batch size per GPU	16 samples (48 total)
Gradient accumulation steps	1 (effective batch size 48)
Number of epochs	30
Total training time	Approximately 12 hours
Maximum sequence length	2048 tokens
Padding strategy	Dynamic padding to longest sequence in batch
Dropout rate	0.1 (applied to attention and feed-forward layers)
Layer Normalization	Applied after each Transformer block
Gradient clipping	Global norm max value set to 1.0
Early stopping	Based on validation loss, the patience of 3 epochs
Parameter initialization	Truncated normal distribution (mean=0, std=0.02) for new parameters
Validation frequency	At the end of each epoch
Model saving strategy	Best model based on validation performance

We employed dynamic padding to the longest sequence in each batch to optimize computational efficiency. Layer Normalization was applied after each Transformer block to stabilize training. For newly introduced parameters, we used a truncated normal distribution (mean=0, std=0.02) for initialization, while retaining pre-trained weights for the base model. To ensure the robustness of our results, all experiments were repeated 10 times with different random seeds. The reported scores represent the mean of these runs, with standard deviations provided in parentheses.

#### Baselines

- **CAML**^15^ is a model that uses a convolutional neural network to encode the document text and a label-wise attention mechanism to generate label-specific representations. It also incorporates the hierarchical structure of the ICD codes to improve the performance of rare labels.
- **MultiResCNN**^20^ is a model that encodes the free text with a multi-filter residual convolutional neural network and applies a label code attention mechanism to enable each ICD code to attend to different parts of the document. It also uses a multi-label focal loss to handle the class imbalance problem.
- **JointLAAT**^41^ is a model that uses a causal meta-learning framework to predict the personalized effects of different interventions. It trains a single meta-model across thousands of tasks, each constructed by sampling an intervention, along with its recipients and nonrecipients. It also uses a hierarchical joint learning mechanism to deal with the imbalanced class issue.
- **KEPTLongformer**^42^ is a model that enhances the Longformer model with medical knowledge from the Unified Medical Language System (UMLS) knowledge graph. It uses a hierarchical self-alignment pre-training method to learn from the hierarchy, synonym, and abbreviation relations in the knowledge graph. It also uses focal loss to improve the performance of rare labels.
- **EffectiveCAN**^13^ is a model that uses a deep convolution-based encoder with squeeze-and-excitation networks and residual networks to aggregate the information across the document and learn meaningful document representations. It also uses multi-layer and sum-pooling attention to extract the most informative features from these representations. It combines binary cross-entropy loss and focal loss to improve performance for rare labels.
- **ICDBigBird**^43^ is a model that employs BigBird^44^, a transformer-based encoder, to deal with the lengthy discharge summary. It leverages Graph Convolutional Network (GCN) to extract the relations between ICD codes and construct contextual ICD embedding.
- **MARN **^27^ is a model that employs multitask learning to facilitate knowledge sharing between different coding branches, enabling the capture of code associations. Furthermore, it includes a recalibrated aggregation module composed of sequential convolutional blocks to extract high-level semantic features, which helps mitigate noise and process lengthy documents effectively.
- **Generation with Prompt (GP)**^30^ is a generative model that generates free-text diagnoses and procedures based on the Subjective, Objective, Assessment and Plan (SOAP) structure, a common medical documentation framework used by physicians. Rather than directly predicting ICD codes, the model generates lower-dimensional text descriptions, which are then used to infer the ICD codes.

**Table 3:** Results on the MIMIC-III-50 and MIMIC-III-FULL compared with baselines.We conducted the experiments on our model 10 times, and each time, we used different random seeds for initialization. We report the mean and standard deviation of each metric.

Model	MIMIC-III-50				MIMIC-III-FULL			
	AUC		F1		AUC		F1	
	Macro	Micro	Macro	Micro	Macro	Micro	Macro	Micro
First-stage classifier								
CAML	87.5	90.9	53.2	61.4	89.5	98.6	8.8	53.9
MultiResCNN	89.9	92.8	60.6	67.0	91.0	98.6	8.5	55.2
JointLAAT	92.5	94.6	66.1	71.6	92.1	98.8	10.7	57.5
EffectiveCAN	92.0	94.5	66.8	71.7	92.1	**98.9**	10.5	58.1
ICDBigBird	90.0	92.9	63.1	69.6	–	–	–	–
MARN	**92.7**	94.7	68.2	71.8	91.3	98.8	11.6	58.4
Reranking-based								
KEPTLongformer	92.6	**94.8**	**68.9**	**72.9**	–	–	11.8	**59.9**
MSMN + GP	–	–	–	–	–	–	**14.6**	59.1
Ours	92.5±0.1	94.6±0.2	67.1±0.1	71.8±0.1	**92.2**±0.2	98.7±0.1	10.7±0.1	58.5±0.1

### Overall performance

We demonstrate the results for the common disease code assignment (MIMIC-III-50) task and the full disease code assignment (MIMIC-III-FULL) task in Table 3. It is observable that our model is comparable to the state-of-the-art model, KEPTLongformer, with only a minor performance gap. However, it is important to note that the KEPTLongformer model is restricted to datasets with precisely 50 labels, limiting its real-world applicability. The reported results on the full disease code assignment task using MIMIC-III-FULL are obtained by first utilizing separate classification models, Multiple Synonyms Matching Network (MSMN)^45^, to reduce the label space to 50 codes, before re-ranking with KEPTLongformer. A similar approach to KEPTLongformer is employed in the GP method, re-ranking the generated ICD codes using a discriminative model on the MIMIC-III-FULL dataset. In contrast, our proposed model is designed for generalized application without constraints on the output space size. Therefore, a direct comparison to KEPTLongformer and GP may be unfair, given the difference in task setting and intended use cases. Compared to the EffectiveCAN and JointLAAT models, which have a similar goal of broad applicability, our approach demonstrates a competitive advantage in performance on the full code assignment task. Moreover, the comparison between our method and ICDBigBird indicates that the proposed pre-trained label tree can better capture the structural relationships of ICD codes than the GCN used in ICDBigBird. Additionally, our method also delivered results similar to those of MARN, the multi-task learning framework, but is more efficient as it does not rely on auxiliary information derived from other tasks during the training process. Overall, while slightly trailing the heavily constrained KEPTLongformer in accuracy, our method achieves state-of-the-art results among models capable of scalable, real-world clinical coding.

### Ablation study

**Table 4:** Ablation study with Deformable Convolution Hidden State Fusion (DCF), Knowledge Graph Embedding (KGE) and Pre-trained Label Tree (PLT).

Model	MIMIC-III-50				MIMIC-III-FULL			
	AUC		F1		AUC		F1	
	Macro	Micro	Macro	Micro	Macro	Micro	Macro	Micro
Ours	92.5±0.1	94.6±0.2	67.1±0.1	71.8±0.1	92.2±0.2	98.7±0.1	10.7±0.2	58.5±0.1
w/o DCF &KGE &PLT	90.2±0.1	92.8±0.1	58.1±0.2	66.6±0.1	90.7±0.1	98.0±0.2	8.1±0.1	54.7±0.1
w/o KGE &PLT	91.3±0.1	93.7±0.1	62.9±0.2	67.8±0.1	91.2±0.1	98.4±0.1	9.1±0.2	56.4±0.1
w/o PLT	91.6±0.1	94.0±0.1	64.4±0.2	68.4±0.1	91.5±0.1	98.5±0.1	9.9±0.2	57.3±0.1

Our proposed model can be characterized as a combination of three architectural components: 1) Deformable Convolution Hidden State Fusion, the fusion of hidden states with 1D deformable convolutional layers; 2) Biomedical Knowledge Integration, injection of external knowledge; and 3) Probabilistic Label Tree: prediction based on a probabilistic label tree. The ablation experiments presented in Table 4 quantify the contribution of each component.

The deformable convolutional layers enable more flexible vector representations compared to standard approaches by adaptively aggregating hidden states. Incorporating external knowledge, such as annotations and ontologies, grounds the model in real-world facts and relationships. Fusing information from multiple modalities with these supplementary cues guides the model to improve predictive performance. Organizing the output space into a probabilistic label tree introduces a structure that captures hierarchies and dependencies among codes. Compared to direct prediction, the tree decoder is better equipped to handle multi-label prediction tasks by modelling inter-code relationships. Without deformable fusion, performance decreases due to less effective integration of the hidden state inputs from multiple encoders. Ablating external knowledge similarly degrades results, as a key inductive bias guiding predictive coherence is removed. Using a flat classifier instead of the tree decoder also impacts accuracy by disregarding output space structure. The combination of all three components—deformable convolutional fusion, external knowledge injection, and the probabilistic label tree decoder—yields the optimal model architecture with improved results.

#### Deformable convolutions for hidden state fusion

A key focus of this work is obtaining effective text representations from pre-trained models. As evidenced in the experimental results, utilizing more dynamic representation techniques can yield more flexible embeddings compared to prevalent approaches like last-first-avg pooling. By emphasizing critical token-level responses during classification, these enhanced text encodings improve performance. Rather than succinctly summarizing the entire sequence, the proposed method generates contextualized representations tailored to each target. Masked pooling selectively focuses on pertinent words, while the deformable convolution adaptively aggregates important fine-grained signals spread throughout the text. In contrast, conventional strategies like last-first-avg produce static condensed embeddings, discarding substantial semantic information. The resulting generic summaries lack the selectivity to highlight relevant local interactions critical for accurate prediction. By extracting representations specialized to each target’s context, our approach better models fine-grained linguistic phenomena while retaining global semantics. The representations distil the most salient signals, providing improved cues for inference compared to fixed summarizations. The gains over established baselines demonstrate the advantages of dynamic context-aware encodings.

#### Injecting external knowledge through multimodal concatenation

This work has two main aims: (1) It looks to emphasize critical keywords by incorporating low-level word vector; (2) Considering the proven vector space differences between knowledge graph embeddings and PLM embeddings, a multimodal concatenation approach is adopted. The results show that this fusion method can improve model performance. Prior work has shown that PLMs largely discard surface word identity and instead encode meaning holistically based on context. Hence, knowledge graph embeddings can retain additional lexical knowledge. The gains over single-source models demonstrate the complementary nature of multimodal encoding. The approach is straightforward yet effective at aggregating diverse feature perspectives. Further work could investigate more sophisticated fusion techniques beyond simple concatenation. Overall, the results highlight the utility of fusing multiple representation types, rather than relying solely on pre-trained features, for enriched encoding. The enhanced embeddings improve downstream performance by consolidating signals from both granular lexical and holistic semantic sources.

#### Probabilistic label tree construction

The probabilistic label tree provides several advantages that improve model performance on multi-label classification tasks. By encoding output dependencies, the tree decoder better handles label co-occurrence and hierarchical relationships compared to flat per-class classifiers. Specifically, the tree incorporates intrinsic structure by grouping related codes into branches, enabling the expression of refined conditional dependencies between labels that independent classifiers miss. This modelling of correlations facilitates the sharing of statistical strengths between related classes and provides useful inductive biases that guide coherent joint predictions based on observed co-occurrence patterns. Additionally, the hierarchical organization propagates signals between coarse-grained parent codes and fine-grained children codes, allowing smoothing of predictions along the directed acyclic graph structure. Computationally, efficiency is improved by pruning unlikely subtrees and beam search, focusing on promising label subsets guided by output space patterns. In summary, by probabilistically modelling output relationships, the tree decoder produces medically coherent predictions aligned with expert knowledge. The explicit encoding of inter-dependencies within the label space allows robust multi-label classification.

## Limitations

While the proposed model achieves strong performance on medical code assignment, certain limitations should be noted: (1) the deformable convolution fusion technique, while more flexible than standard approaches, may still lack the representational power to fully capture complex semantic interactions. More advanced fusion methods could be explored; (2) the external knowledge injection is limited to the basic concatenation of supplementary features. Investigating learned integration or attention mechanisms may further enhance multimodal synergy; (3) the label tree decoder imposes an intrinsic output structure that may not fully align with the optimal data-driven dependencies. Allowing end-to-end learning of output relationships could potentially improve results. Inference is also slowed by requiring sequential tree decoding.

## Conclusion and future work

Overall, this work presents a novel approach for automated ICD coding of clinical text, combining deformable convolutional fusion, external knowledge injection, and probabilistic label tree decoding. The results demonstrate state-of-the-art performance on the MIMIC-III benchmark, validating the effectiveness of the proposed techniques. However, limitations exist that could be addressed in future work: (1) The representation fusion could be enhanced with more sophisticated deep learning approaches; (2) Multimodal integration could also utilize mechanisms that are learned beyond basic concatenation; (3) Finally, directly learning the output space dependencies in an end-to-end manner could potentially improve upon the predefined tree structure.

## Data Availability

All data supporting this research are from previously reported studies and datasets, which have been cited within the article. The processed data are available from the corresponding author upon request.
